# Temporal windows of unconscious processing cannot easily be disrupted

**DOI:** 10.1167/jov.24.4.21

**Published:** 2024-04-24

**Authors:** Lukas Vogelsang, Leila Drissi-Daoudi, Michael H. Herzog

**Affiliations:** 1 Laboratory of Psychophysics, École Polytechnique Fédérale de Lausanne (EPFL), Lausanne, Switzerland

**Keywords:** temporal integration, unconscious processing, windows of integration, sequential metacontrast paradigm

## Abstract

Conscious perception is preceded by long periods of unconscious processing. These periods are crucial for analyzing temporal information and for solving the many ill-posed problems of vision. An important question is what starts and ends these windows and how they may be interrupted. Most experimental paradigms do not offer the methodology required for such investigation. Here, we used the sequential metacontrast paradigm, in which two streams of lines, expanding from the center to the periphery, are presented, and participants are asked to attend to one of the motion streams. If several lines in the attended motion stream are offset, the offsets are known to integrate mandatorily and unconsciously, even if separated by up to 450 ms. Using this paradigm, we here found that external visual objects, such as an annulus, presented during the motion stream, do not disrupt mandatory temporal integration. Thus, if a window is started once, it appears to remain open even in the presence of disruptions that are known to interrupt visual processes normally. Further, we found that interrupting the motion stream with a gap disrupts temporal integration but does not terminate the overall unconscious processing window. Thus, while temporal integration is key to unconscious processing, not all stimuli in the same processing window are integrated together. These results strengthen the case for unconscious processing taking place in windows of sensemaking, during which temporal integration occurs in a flexible and perceptually meaningful manner.

## Introduction

Conscious perception is substantially delayed. We have argued that conscious percepts are preceded by extended periods of unconscious processing (Drissi-Daoudi, Doerig, & Herzog, 2019; Herzog, Drissi-Daoudi, & Doerig, 2020; Herzog, Kammer, & Scharnowski, 2016; Otto, Ögmen, & Herzog, 2006). These periods are required, for instance, to detect and process motion signals and to integrate even static information in challenging perceptual situations. Consider the scenario of a car moving through the night, with its surface catching reflections from the streetlights and its path being occluded by other vehicles and objects. Such circumstances make it challenging to infer certain details, such as the color of the car, from the activity of individual photoreceptors. To do so more reliably, the activity of photoreceptors may be averaged over the course of the car's motion trajectory.

Considering their relevance for perceptual function, it is important to carefully examine the mechanisms of temporal windows of unconscious processing. However, most experimental paradigms do not offer the methodology required for such investigations. This is because stimuli are usually presented for short times, and even when presented for long intervals, there is no mandatory integration, which is needed to study the duration of processing. The sequential metacontrast paradigm (SQM) has turned out to be a well-suited psychophysical tool for this purpose, as it allows measuring mandatory and unconscious visual feature integration in a systematic and flexible manner (Drissi-Daoudi et al., 2019; Drissi-Daoudi, Öğmen, Herzog, & Cicchini, 2020; Drissi-Daoudi, Öğmen, & Herzog, 2021; Menetrey, Herzog, & Pascucci, 2023; Otto et al., 2006; Otto, Ögmen, & Herzog, 2009; Otto, Ögmen, & Herzog, 2010; Plomp, Mercier, Otto, Blanke, & Herzog, 2009; Scharnowski et al., 2009; Vogelsang, Drissi-Daoudi, & Herzog, 2023). Here, we used the SQM to examine how unconscious processing windows are started and what may disrupt them.

**Figure 1.**
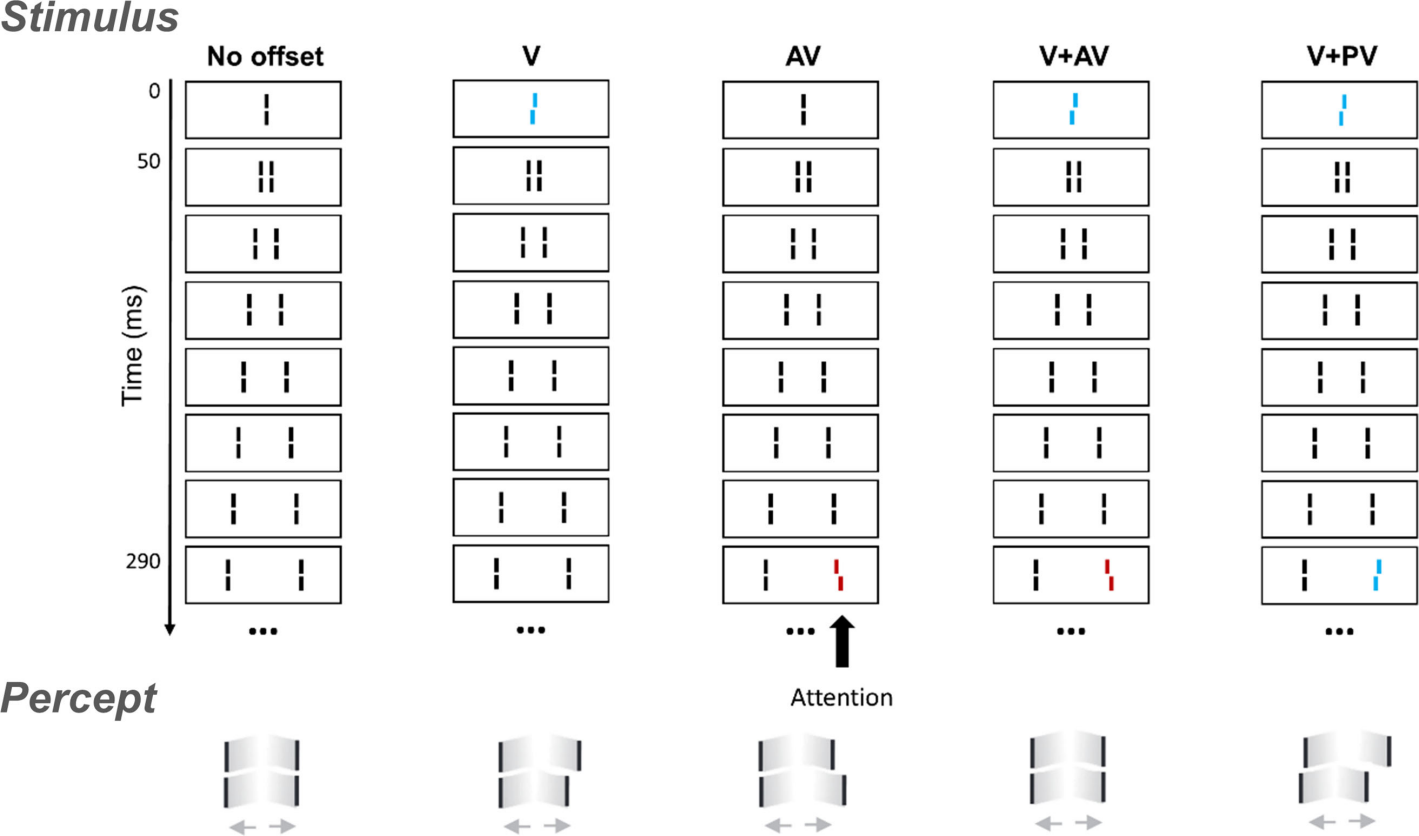
In the SQM, a central line is initially presented, followed by pairs of flanking lines. This elicits the perception of two motion streams diverging from the center. Participants are asked to direct their attention to one of the motion streams (in this illustration, the right one) and to report the perceived offset direction. When one of the lines in the attended stream is offset, the entire stream appears to be offset, even though the other lines are actually straight. If both the central and a later flanking line have an offset, the two offsets integrate, if separated by less than approximately 450 ms (Drissi-Daoudi et al., 2019). Specifically, if the two offsets are in opposite directions, they effectively cancel each other out before reaching consciousness. If, instead, the two offsets are in the same direction, performance improves. The colors used here are only for illustration. In the actual experiment, all lines were white, presented on a black background. This illustration is adapted from Drissi-Daoudi et al. (2019) and Vogelsang et al. (2023).

In the SQM (Otto et al., 2006), participants are shown a central line followed by pairs of flanking lines, resulting in the perception of two diverging motion streams originating from the center (see Figure 1). The central line is invisible due to metacontrast masking. However, when the central line is offset (referred to as “vernier” offset or V, where the lower segment of the line is shifted to the left or the right, relative to the upper segment), participants perceive the subsequent flanking lines as offset, even though these lines are actually straight. When one of the flanking lines is also offset, the two offsets integrate. If both offsets are in the same direction (“pro-vernier” or PV), performance improves. If the two offsets are in opposite directions (“anti-vernier” or AV), they cancel each other out before reaching conscious awareness, even when separated by up to 450 ms (Drissi-Daoudi et al., 2019). In this case, observers cannot report the individual vernier offsets independently. Hence, integration in the SQM is mandatory. Further, when different offsets are presented at 0 ms, 290 ms, and 450 ms, the first two offsets integrate, but the third one can be reported independently. Thus, instead of following spatiotemporal proximity, integration appears to occur in discrete temporal windows of processing, starting with stimulus onset (Drissi-Daoudi et al., 2019).

**Figure 2.**
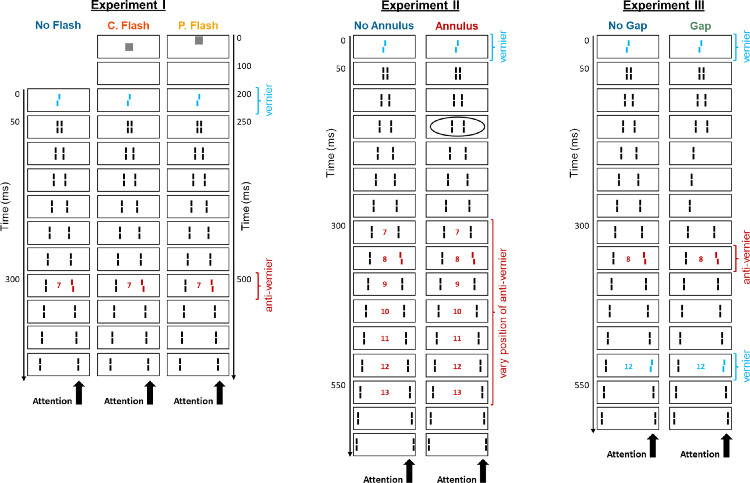
Illustration of the three SQM experiments. In the “flash” conditions of Experiment 1, a flash was presented prior to the onset of the typical SQM sequence, either in the center of the screen (“central flash” condition) or 60′ above the center (“peripheral flash” condition). In the “annulus” condition of Experiment 2, an annulus (ellipse) was shown during the presentation of the third flanking line. In the “gap” condition of Experiment 3, the lines of Frames 4–6 in the attended stream were not presented. The colors in this figure are only used for illustration. In the real experiment, all lines were displayed in white, on a black background. The frame numbers depicted in the figure are only for illustrative purposes and were not shown in the real experiment.

In this article, we report three experiments with the SQM, aimed at examining how unconscious windows of processing are started (Experiment 1) and what may disrupt them (Experiments 2 and 3). The three experiments are illustrated in Figure 2. In our first experiment, we presented a flash prior to the SQM stimulus sequence, and we tested whether or not it would start a global processing window that is still encompassing offsets presented during the SQM motion stream. Specifically, following the (pre-SQM) flash, we presented a central vernier and a later anti-vernier at Frame 7, and participants had to report the latter offset. The rationale is that if the flash starts a window of processing, and if the onset of the motion stream does not start a new window of processing, then the initial vernier would fall into the first processing window, while the anti-vernier at Frame 7 would be in the second window. Thus, integration should be reduced, compared to integration in a no-flash condition.

In the second experiment, we presented an annulus during the motion stream, in addition to a central vernier and a later anti-vernier. The rationale here is that if the annulus were to disrupt processing, the vernier and anti-vernier should integrate less, compared to a nonannulus condition. Finally, in Experiment 3, we presented a gap during the stream (at Frames 4 through 6), in addition to a vernier at Frame 0, an anti-vernier at Frame 8, and a pro-vernier at Frame 12. Participants were asked to report the final offset direction. Here, the rationale is that, without a gap, participants should be able to report the final vernier, as the offsets at positions 0 and 8 fall into the first window of processing, and the final offset at Frame 12 falls into a second window of processing (Drissi-Daoudi et al., 2019). However, if the gap were to disrupt the processing window and start a new one, then the offsets presented at Frames 8 and 12 should fall into the same integration window, and the ability to report the final offset should thus be lower.

## Methods

### Participants

In this study, naive participants were enlisted from the École Polytechnique Fédérale de Lausanne and the University of Lausanne in Switzerland. Experiment 1 was successfully completed by 16 participants (6 females; age range: 19–24 years), Experiment 2 by 8 participants (2 females; age range: 19–27 years), and Experiment 3 by 12 participants (2 females; age range: 19–26 years). Before the experiment, participants provided informed consent. All individuals had normal or corrected-to-normal vision, as indicated by an acuity of at least 1.0 binocularly on the Freiburg Visual Acuity test (Bach, 1996). Upon conclusion of the study, participants were compensated monetarily. The experiments and procedures adhered to the principles of the Declaration of Helsinki, with the exception of preregistration, and were approved by the local ethics committee (Commission cantonale d'ethique de la recherche sur l'etre humain) of canton Vaud in Switzerland.

### Apparatus

The visual stimuli were presented on a BenQ XL2540 LCD monitor (24.5 in., 1,920 × 1,080 pixels, 240 Hz). MATLAB R2013a (MathWorks, Natick, MA, USA) and Psychtoolbox (Brainard, 1997) were used to generate the stimuli. Participants were seated 2.5 m from the screen in a room that was dimly lit. The stimuli were presented in white (100 cd/m²) on a black background.

### Stimuli

After the presentation of a fixation dot lasting 0.5 s, followed by a 0.5-s blank screen, the standard SQM stimulus sequence was presented. First, a central line composed of both an upper and a lower line segment, each measuring 26.5′ (arcmin) in length and separated vertically by a gap of 2.3′, was presented. Following the central line, pairs of flanking lines, matching the length and vertical separation of the central line, were presented one pair at a time, appearing progressively farther away from the center. Individual lines were 1.2′ wide and horizontally spaced 3.5′ apart. Each line was displayed for a duration of 21 ms. While the interstimulus interval (ISI) between the central and the first flanking line was 29 ms (in order to ensure strong masking of the central vernier), the ISI between subsequent flanking lines was 21 ms. This stimulus sequence led to the perception of two motion streams diverging from the center toward the periphery.

Participants were instructed to covertly focus their attention on one of the motion streams (here, the right stream). In all experiments reported here (see Figure 2 for illustration of the three specific experiments), the central line presented at Frame 0 was offset, meaning that the lower line segment was shifted to the left or the right, relative to the upper segment. The line with this offset is referred to as “vernier” or V. In addition, one of the subsequent flanking lines in the attended stream was offset in the opposite direction, referred to as “anti-vernier” or AV. Note that in this standard SQM configuration, if vernier and anti-vernier are presented within the first approximately 450 ms, participants perceive only a single, integrated offset, regardless of the number of offsets presented. Participants cannot respond to the individual offsets. Thus, integration is mandatory (Drissi-Daoudi et al., 2019).

The participants’ task was to indicate whether they perceived a left or right offset at the end of the stimulus stream (see the paragraphs to follow for details). After the stimulus presentation, participants had 3 s to respond by clicking one of two handheld buttons. The subsequent trial started 0.5 s after the response. Auditory feedback for wrong responses was provided during the calibration phase (see section on calibration below) but not during the main experiment (except for specific parts of Experiment 2, as detailed further below). Illustrations of the different stimulus configurations used in the three experiments presented in this article can be found in Figure 2.

#### Experiment 1

In the “no-flash” condition of Experiment 1, a central vernier at Frame 0 was followed by an anti-vernier at Frame 7. The “flash” conditions were identical to the “no-flash” condition, except that a white square of width 26.5′ was presented in the center (“central flash” condition) or 60′ above the center (“peripheral flash” condition) for 100 ms, followed by a 100-ms blank screen. A total of 10 flanking lines was presented in both conditions. Participants were asked to report the offset they perceived at the end of the attended stream.

#### Experiment 2

In the “no-annulus” condition of Experiment 2, a central vernier was followed by an anti-vernier, whose position was randomly determined to be between Frames 7 and 13. In the “annulus” condition, an annulus was displayed during the presentation of Frame 3, with a luminance of 40%, a radius of 120′, and a width of 25′. There was a total of 15 flanking lines (the last one presented at 632 ms), and participants were instructed to report the offset they perceived at the end of the attended stream.

#### Experiment 3

In the “no-gap” condition of Experiment 3, a central vernier at Frame 0 was followed by an anti-vernier at Frame 8 and a pro-vernier (i.e., an offset in the same direction as the central vernier offset) at Frame 12. In the “gap” condition, the lines of Frames 4–6 of the attended stream were not presented. In this experiment, participants were informed about the presentation of three different offsets and were asked to report the direction of the last offset. As detailed further below, for some of the tested blocks, auditory feedback has been provided for wrong responses. There was a total of 14 flanking lines.

### Offset size calibration

Prior to the main experiment, we calibrated the offset sizes (i.e., the displacement between the two line segments) for each relevant offset position. This was done to ensure that the different offsets presented in the SQM are equivalent in terms of their individual perceptual impact. Given the variability between participants, this calibration process was carried out not only for each relevant position but also for each individual participant separately. To this end, sequences featuring only a single offset at a specific position were presented, and an adaptive parameter estimation by sequential testing (PEST) procedure (Taylor & Creelman, 1967) was utilized to determine the offset sizes necessary to achieve performance levels of 75%.

In Experiment 1, offset sizes were calibrated for Frames 0, 3, and 7, respectively. In Experiment 2, offset sizes were calibrated for Frames 0 and 10, respectively. As in Vogelsang et al. (2023), the offset at Frame 10 was used as a proxy for all offset positions presented later (positions 7–13). In Experiment 3, offset sizes were calibrated for Frames 0, 8, and 12, respectively. In all three experiments, the offset sizes were calibrated only with the standard conditions but were later retested with the modified conditions (i.e., the annulus, gap, and flash conditions). The means and standard deviations of calibrated offset sizes (in arcseconds) were as follows: 96.9 ± 45.8 (Experiment 1, vernier position 0), 67.8 ± 28.0 (Experiment 1, vernier position 3), 46.9 ± 13.1 (Experiment 1, vernier position 7), 66.3 ± 26.7 (Experiment 2, vernier position 0), 43.8 ± 9.2 (Experiment 2, vernier position 10), 122.1 ± 51.2 (Experiment 3, vernier position 0), 78.8 ± 33.9 (Experiment 3, vernier position 8), and 52.9 ± 21.5 (Experiment 3, vernier position 12).

### Experimental procedure

The main conditions measured for each of the three experiments are depicted in Figure 2. The very specifics of the experimental sessions are detailed in this section.

In Experiment 1, participants completed 21 blocks of 80 trials each. The 21 blocks were split into three parts. In Part 1 (nine blocks), participants were presented with a single vernier, at Frame 0, 3, or 7, and in combination with no flash, a central flash, or a peripheral flash. In Parts 2 and 3 (six blocks each), participants were presented with a central vernier that was followed by an anti-vernier at Frame 3 or 7 and with no flash, a central flash, or a peripheral flash. The order of blocks within each part was randomized.

In Experiment 2, following the calibration phase, participants completed 18 blocks of 90 trials each. The blocks were presented in the following order: NAANANNAANNANAANAN, with “N” representing the nonannulus condition and “A” representing the annulus condition. Each block contained 10 trials for each of nine different conditions, with the 90 trials randomized for each block and participant. The nine tested conditions comprised seven conditions in which two opposing offsets appeared (V0 + AV7, V0 + AV8, V0 + AV9, V0 + AV10, V0 + AV11, V0 + AV12, V0 + AV13) as well as two control conditions in which only a single vernier was presented (V0-alone, V10-alone).

In Experiment 3, following the calibration phase, participants completed 16 blocks of 80 trials each. These 16 blocks were split into three parts. In the first three blocks of Part 1, participants were presented with a motion stream with three offsets (a vernier offset at Frame 0, an anti-vernier offset at Frame 8, and a pro-vernier offset at Frame 12) and received auditory feedback for wrong responses. Participants were informed about the presence of three different offsets being presented and were asked to report the direction of the third offset. In the fourth block, the same stimuli were presented as in the first three blocks, but no response feedback was provided. In the fifth block, no response feedback was provided, and as an additional control, the direction of the second and third offsets flipped (resulting in the presentation of a central vernier followed by a pro-vernier at Frame 8 and an anti-vernier at Frame 12). It was randomly determined in which condition a given participant would complete these five blocks (i.e., in the “gap” or “no-gap” condition). Part 1 was identical to Part 2, except that stimuli were presented for the opposite condition (i.e., the “no-gap” or “gap” condition). Finally, in Part 3, participants completed six conditions, which were presented in random order. They comprised the presentation of a single vernier that was presented at Frame 0, 8, or 12 and either with or without the gap. Here, participants were instructed to report the direction of the offset that they perceived at some point in the attended motion stream.

### Data analysis

For each experiment and condition, we extracted the initial (or final) vernier dominance level, that is, the fraction of responses that was in accordance with the direction of the initial (or final) vernier offset that was presented in the attended stream. For instance, if a central vernier and a later anti-vernier are presented, an anti-vernier dominance (or “final vernier dominance”) of 0% indicates that all responses were made in agreement with the offset direction of the initial vernier. An anti-vernier dominance of 100% indicates that all responses were aligned with the direction of the anti-vernier, and an anti-vernier dominance of 50% indicates that responses were equally often aligned with the central vernier and the later anti-vernier offset direction.

For each of the three experiments, we extracted the main condition of interest across the experimental variations (i.e., central flash, peripheral flash, and no flash in Experiment 1; annulus and no annulus in Experiment 2; and gap and no gap in Experiment 3). Subsequently, these were compared with a repeated-measures analysis of variance (ANOVA) (in Experiment 1) and paired *t*-tests (in Experiments 2 and 3).

Data analysis was performed using MATLAB R2022b (MathWorks).

### Power analysis

The SQM (Otto et al., 2006) has a large effect size, with a Cohen's *d* of usually around 1.5 or greater (Drissi-Daoudi et al., 2019; Drissi-Daoudi et al., 2020). With this effect size, a modest sample size of only seven participants would be sufficient for achieving a power greater than 90% when applying a paired-sample two-tailed *t*-test with an error probability of 0.05. However, to be on the safe side, we recruited at least 8 participants for each experiment (16 for Experiment 1, 8 for Experiment 2, and 12 for Experiment 3). More participants were thereby recruited for Experiments 1 and 3 due to a slightly more complex experimental design. This is because, in Experiment 1, three different experimental conditions were tested and compared using an ANOVA rather than a *t*-test, and in Experiment 3, three offsets were simultaneously present, which could potentially increase response noise.

## Results

### A flash before the SQM does not create a global unconscious processing window that encompasses offsets in the motion stream

In Experiment 1, a central vernier and an anti-vernier at Frame 7 were shown, and participants were instructed to report the latter offset. In the “flash” condition, a flash was presented prior to the SQM (see Figure 2 for illustration).

**Figure 3.**
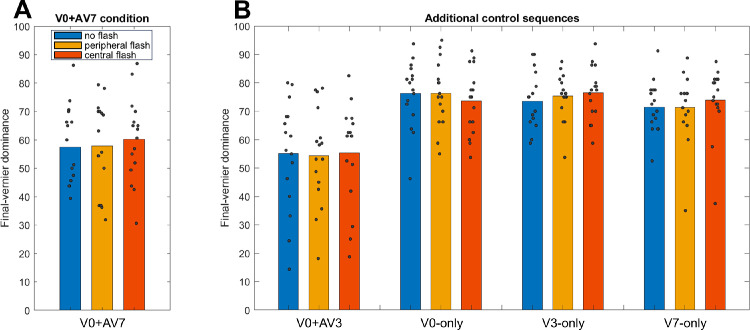
(**A**) Vernier dominance of the V0 + AV7 sequence. The vernier and anti-vernier integrate in all three conditions, and dominance does not vary significantly across conditions. (**B**) Vernier dominance for the V0 + AV3, V0-alone, V3-alone, and V7-alone sequences, as additional controls. Dominance levels are similar across conditions. Gray dots represent individual data points.

In the normal (i.e., “no-flash”) condition, the two offsets should integrate, and participants should not be able to report them individually (Drissi-Daoudi et al., 2019). However, if the flash were to start a window of processing and the onset of the SQM were to not start a new window of processing, then the vernier and anti-vernier should not integrate anymore, and the vernier dominance should decrease. However, this is not what we observed. As depicted in Figure 3A, in all three conditions (no flash, central flash, peripheral flash), observers were not able to report the anti-vernier offset. Dominance in the three conditions was comparable (*F*(2, 30) = 0.406, *p* = 0.670, η^2^ = 0.026 in one-way repeated-measures ANOVA comparing dominance in the V0 + AV7 sequence across the three conditions). Thus, the window of processing started by the flash appears to no longer be open when the SQM motion stream begins. Alternatively, the processing window created by the flash could be far shorter (i.e., ending before the central vernier) or much longer (i.e., encompassing both vernier and anti-vernier) than normal processing windows in the SQM. This is elaborated on further in the Discussion section.

Figure 3B also depicts dominance levels for additional control sequences. These dominance levels, too, are similar across conditions.

### An annulus presented during the motion stream does not disrupt integration

In Experiment 2, a central vernier and a later anti-vernier were presented, and participants were asked to report the perceived offset direction at the end of the motion stream. In the “annulus” condition, an annulus was additionally presented at Frame 3 (see Figure 2 for illustration).

**Figure 4.**
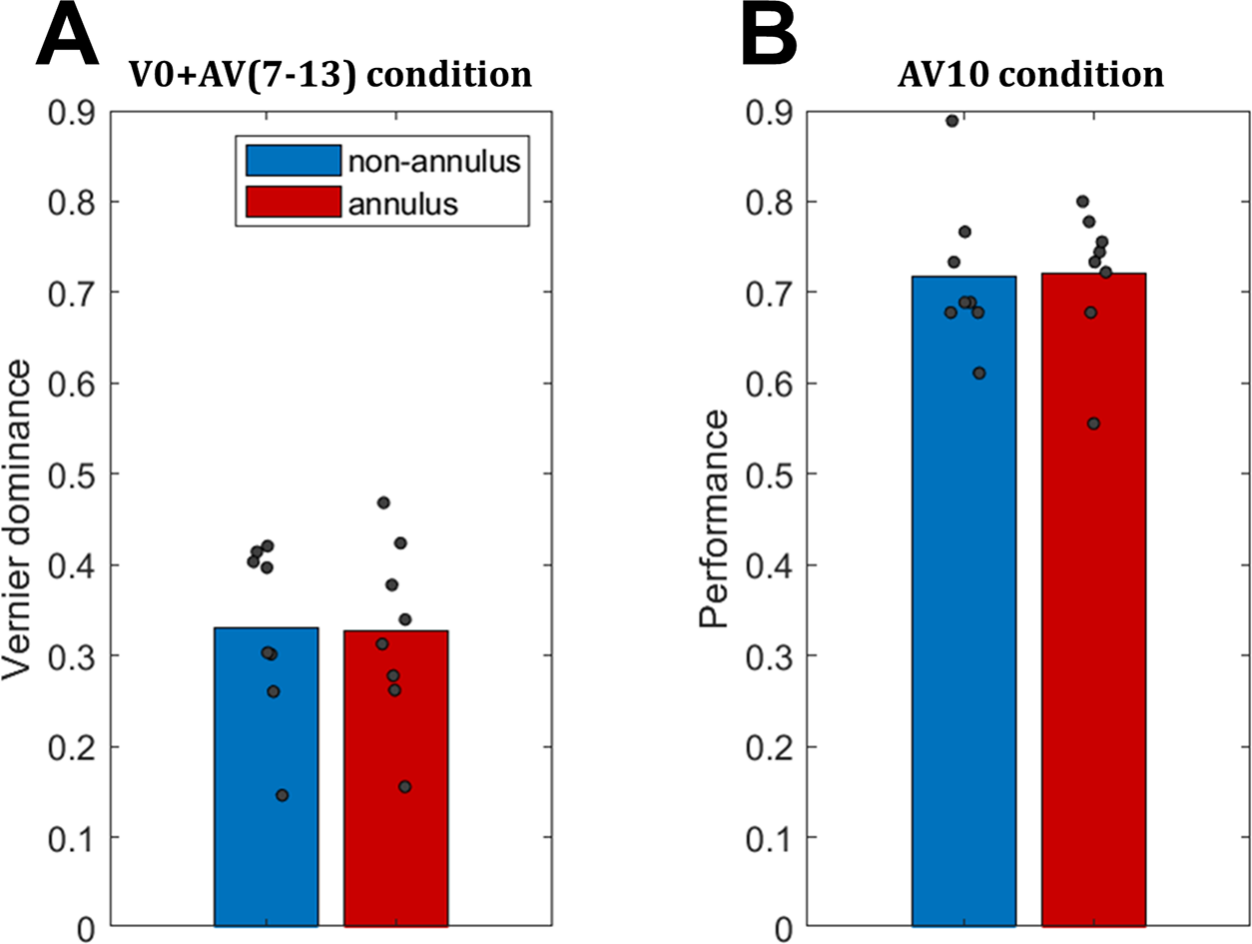
(**A**) Vernier dominance in the V + AV condition, averaged across the frames at which the anti-vernier appeared (Frames 7–13). As the dominance is almost identical across conditions, the annulus does not disrupt integration. (**B**) Control condition in which only a single vernier at position 10 was presented. The ability to report the single offset in both conditions is similar. Gray dots represent individual data points.

If the annulus were to disrupt integration between the vernier and anti-vernier, observers should be better at reporting the direction of the anti-vernier, relative to the nonannulus condition. Thus, the vernier dominance should be lower. However, this is not what we found. As Figure 4A shows, the vernier dominance was almost identical across the two conditions (*t*(7) = 0.308, *p* = 0.767, Cohen's *d* = 0.109 in two-tailed paired-sample *t*-test comparing mean dominance in the V + AV sequences across both conditions). Thus, the annulus does not disrupt integration. As an additional control, Figure 3B shows that the ability to report the offset direction of a single vernier at position 10 is also comparable across the two conditions.

### A gap presented during the motion stream interrupts integration but does not terminate the entire unconscious processing window

In Experiment 3, a vernier at Frame 0, an anti-vernier at Frame 8, and a pro-vernier at Frame 12 were presented, and participants were instructed to report the direction of the third offset. In the “gap” condition, the lines at Frames 4 through 6 in the attended stream were rendered invisible (see Figure 2).

Normally, observers are able to report the final pro-vernier at Frame 12, as it falls into a different temporal window than the earlier vernier (at Frame 0) and anti-vernier (at Frame 8) (Drissi-Daoudi et al., 2019). If the gap were to disrupt the entire window of processing and start a new one, then observers should not be able to report the pro-vernier, as the pro-vernier would fall into the same window as the anti-vernier at Frame 8.

**Figure 5.**
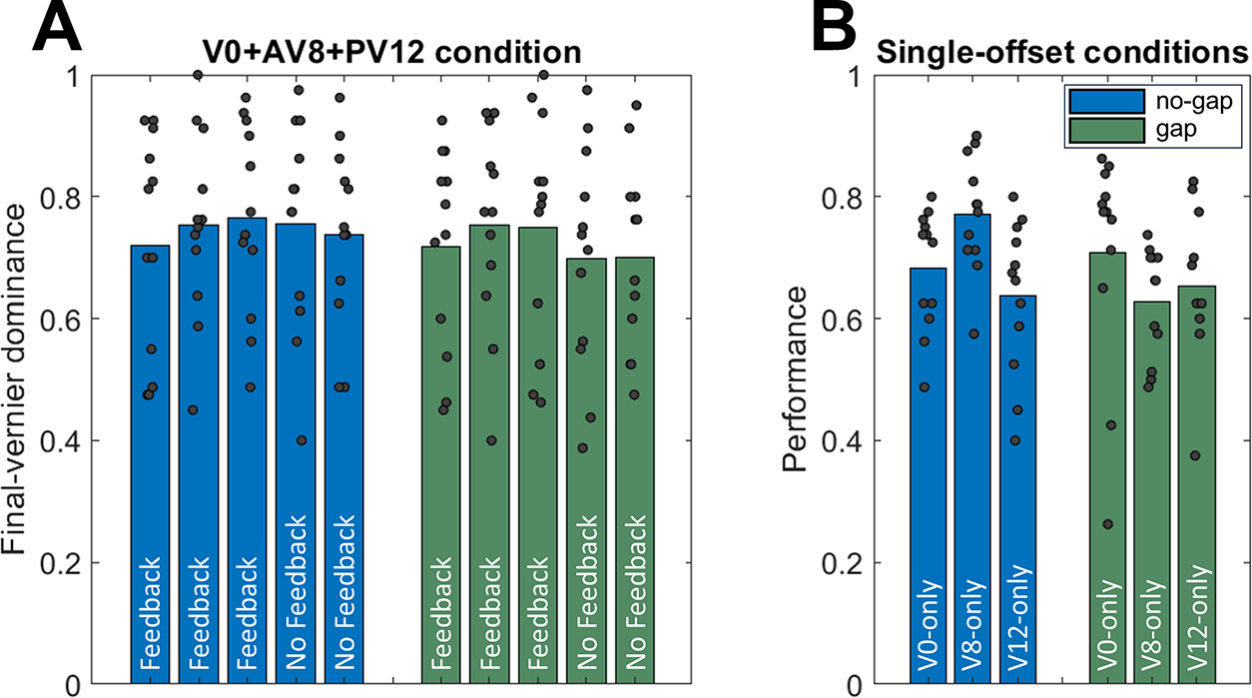
(**A**) Dominance with regard to the final vernier presented. The first three blocks of each condition are with feedback. The fourth and fifth blocks are without feedback, and the fifth block had flipped offset directions for offsets 2 and 3. As the results are comparable across conditions, the gap does not terminate the window of processing. (**B**) Performance of reporting the offset directions in streams with single verniers at positions 0, 8, and 12 for each of the two conditions. Gray dots represent individual data points.

As depicted in Figure 5A, both with and without feedback, observers were able to report the final pro-vernier, with dominance in the two conditions being comparable (*t*(11) = 1.204, *p* = 0.254, Cohen's *d* = 0.348 in two-tailed paired-sample *t*-test comparing the mean of the five blocks of the V + AV + PV sequences across the two conditions). Thus, the gap does not disrupt windows of processing.

Importantly, the gap is known to disrupt integration between the central vernier and the later anti-vernier (Drissi-Daoudi et al., 2020). Thus, even though the gap disrupts integration, it does not terminate the entire window of processing. This highlights the important difference between the two, which is further elaborated on in the Discussion.

As additional controls, Figure 5B depicts the performance in vernier-only sequences for both conditions. The results are fairly similar across conditions, with only relatively minor deviations.

## Discussion

Windows of unconscious processing, preceding conscious awareness, are crucial for visual perception (though for discussion, see Doerig, Scharnowski, & Herzog, 2019; Fekete, Van de Cruys, Ekroll, & van Leeuwen, 2018; Hogendoorn, 2022). In the SQM, these windows last up to around 450 ms. Notably, long integration periods have also been reported in other experimental paradigms. For instance, it was shown that the introduction of a cue up to 400 ms after a visual stimulus can alter the perception of that stimulus (Sergent, 2018; Sergent et al., 2013; Thibault, Van den Berg, Cavanagh, & Sergent, 2016). Similarly, studies using Rapid Serial Visual Presentation (RSVP) paradigms demonstrated that integration can persist for more than 200 ms (Akyürek & Wolff, 2016). Here, we have examined what starts these windows and how they may be disrupted.

In our first experiment, by presenting a flash before the SQM stimulus sequence, we set out to study what starts unconscious windows of processing. The results reveal that the flash does not start a global window of processing that remains active and encompasses the central offset in the SQM motion stream. Instead, it appears that the onset of the SQM starts a new window of processing. We did not expect this result as the other experiments reported here indicate that once a processing window is active, it cannot easily be disrupted. However, it is important to note that an alternative explanation exists for these findings. It is possible that the duration of the processing window, started by the flash, is very different from the typical SQM window durations of 450 ms. We have shown in the past that the integration duration in the SQM is not fixed but depends on factors such as the processing load (Vogelsang et al., 2023). If the window comprising the flash were far shorter than 450 ms, then the window would have ended, without disruption, already prior to the presentation of the stimulus stream. Alternatively, if the window comprising the flash was much longer than 450 ms, then the window would encompass both the central vernier and later anti-vernier. In either of these cases, the vernier and anti-vernier would integrate, just as they do in the reported experiments. Thus, we cannot exclude this possibility.

In general, as argued in Herzog et al. (2020), it is important to note that it is difficult to experimentally determine temporal integration windows directly. For instance, if the detection of nonsimultaneous events occurs at a certain temporal resolution, it does not allow us to infer that conscious percepts occur at this resolution. Instead, these experiments rather measure the temporal resolution of an unconscious feature detector, for example, for simultaneity, which precedes consciousness logically. Temporal parameters of consciousness per se can only be estimated, for instance, by postdictive effects, such as in the SQM.

Our second experiment revealed that an annulus presented during the motion stream does not disrupt temporal integration. This is in line with the finding that occluders superimposed on the attended motion stream—thereby hiding the moving line for some part of the motion trajectory—do not disrupt integration between the offsets presented before and after the occluder (Drissi-Daoudi et al., 2021). Together, both of these findings support the proposal that object identity determines temporal integration (Drissi-Daoudi et al., 2021). Thus, both the occluder and the annulus appear to be detected as objects that exist independent of the motion stream and, therefore, do not affect the integration of the motion stream itself. Even eye movements, which provide a strongly disruptive signal for the retina, do not lead to the disruption of temporal integration (Drissi-Daoudi et al., 2020). That is, offsets presented before and after participants were asked to make a saccade still integrated mandatorily, despite this strong change in the retinal image. Thus, integration is remarkably stable and depends on high-level processing rather than on low-level signals.

Finally, further building up on these findings, in our third experiment, we examined the effect of a gap that was present during the motion stream. Drissi-Daoudi et al. (2021) previously revealed that interrupting the motion stream using a gap disrupts mandatory integration between the offsets presented before and after the gap. This appears perceptually meaningful as, unlike in the case of the occluder or annulus, which can be interpreted as objects that are distinct from the motion stream, the presence of a gap disrupts the object integrity of the motion stream. We fruitfully extend this finding by showing that while the gap disrupts integration, it does not disrupt the entire processing window. That is, the segments of the motion stream before and after the gap appear to be processed as two visual objects, which are not integrated together but are nevertheless part of the same unconscious processing window. Hence, while temporal integration is a key component of unconscious processing, not all stimuli in the same processing window are integrated together. This makes sense as not all stimuli presented within the same window of processing *should* be integrated. For instance, if two cars are driving in different directions, their motion trajectories should not be integrated together. Nevertheless, the cars should be processed as part of the same window of processing (or “sensemaking”). These results thus highlight the important difference between integration and processing windows: It is possible that mandatory integration is disrupted but that the overall window of processing (in which many, but not all, features are integrated) is not.

Future experiments could further strengthen the case made in the current study. For instance, it would be interesting to examine whether the effect of, for example, the annulus would be similarly low if observing the annulus were task-relevant. This could be tested, for instance, in a dual-task experiment, where a certain feature of the annulus, such as its color, has to be reported, in addition to the offset direction. Furthermore, we cannot exclude the possibility that visual observers got used to the “normal” SQM sequence as part of the calibration phase (where no annulus, flash, or gap was shown). Therefore, it would be interesting to examine whether participants would respond differently had they never been exposed to the nonmanipulated version of the SQM first. Finally, carrying out SQM experiments with response modes that exceed binary decisions would be especially insightful in order to examine the phenomenology and underlying mechanisms of spatiotemporal integration. For instance, a “no response” option or even continuous response options could be added, in addition to, potentially, confidence ratings.

Taken together, the main conclusion deriving from this article is that once windows of processing, started by the SQM stimulus onset, are active, they stay active. Even if certain stimulus variations may cause a disruption of temporal integration, they do not necessarily cause a disruption of the processing window. Overall, the experiments reported here thus strengthen the case for unconscious processing taking place in windows of sensemaking, during which temporal integration occurs in a flexible and rather high-level manner.

## References

[bib1] Akyürek, E. G., & Wolff, M. J. (2016). Extended temporal integration in rapid serial visual presentation: Attentional control at Lag 1 and beyond. *Acta Psychologica,* 168, 50–64, 10.1016/j.actpsy.2016.04.009. 27155801

[bib2] Bach, M. (1996). The Freiburg Visual Acuity Test—Automatic measurement of visual acuity. *Optometry and Vision Science,* 73(1), 49–53, 10.1097/00006324-199601000-00008. 8867682

[bib3] Brainard, D. H. (1997). The psychophysics toolbox. *Spatial Vision,* 10(4), 433–436, https://psycnet.apa.org/doi/10.1163/156856897X00357. 9176952

[bib4] Doerig, A., Scharnowski, F., & Herzog, M. H. (2019). Building perception block by block: A response to Fekete et al. *Neuroscience of Consciousness,* 2019(1), niy012, 10.1093/nc/niy012. 30723552 PMC6349944

[bib5] Drissi-Daoudi, L., Doerig, A., & Herzog, M. H. (2019). Feature integration within discrete time windows. *Nature Communications,* 10(1), 1–8, 10.1038/s41467-019-12919-7. PMC6814726 31653844

[bib6] Drissi-Daoudi, L., Öğmen, H., Herzog, M. H., & Cicchini, G. M. (2020). Object identity determines trans-saccadic integration. *Journal of Vision,* 20(7), 33, 10.1167/jov.20.7.33. 32729906 PMC7424110

[bib7] Drissi-Daoudi, L., Öğmen, H., & Herzog, M. H. (2021). Features integrate along a motion trajectory when object integrity is preserved. *Journal of Vision,* 21(12), 4, 10.1167/jov.21.12.4. PMC8572464 34739035

[bib8] Fekete, T., Van de Cruys, S., Ekroll, V., & van Leeuwen, C. (2018). In the interest of saving time: A critique of discrete perception. *Neuroscience of Consciousness,* 2018(1), niy003, 10.1093/nc/niy003. 30042856 PMC6007149

[bib9] Herzog, M. H., Drissi-Daoudi, L., & Doerig, A. (2020). All in good time: Long-lasting postdictive effects reveal discrete perception. *Trends in Cognitive Sciences,* 24(10), 826–837, 10.1016/j.tics.2020.07.001. 32893140

[bib10] Herzog, M. H., Kammer, T., & Scharnowski, F. (2016). Time slices: What is the duration of a percept? *PLoS Biology,* 14(4), e1002433, 10.1371/journal.pbio.1002433. 27070777 PMC4829156

[bib11] Hogendoorn, H. (2022). Perception in real-time: Predicting the present, reconstructing the past. *Trends in Cognitive Sciences,* 26(2), 129, 10.1016/j.tics.2021.11.003. 34973925

[bib12] Menétrey, M. Q., Herzog, M. H., & Pascucci, D. (2023). Pre-stimulus alpha activity modulates long-lasting unconscious feature integration. *NeuroImage,* 278, 120298, 10.1016/j.neuroimage.2023.120298. 37517573

[bib13] Otto, T. U., Ögmen, H., & Herzog, M. H. (2006). The flight path of the phoenix—The visible trace of invisible elements in human vision. *Journal of Vision,* 6(10), 7, 10.1167/6.10.7. 17132079

[bib14] Otto, T. U., Ögmen, H., & Herzog, M. H. (2009). Feature integration across space, time, and orientation. *Journal of Experimental Psychology: Human Perception and Performance,* 35(6), 1670, https://psycnet.apa.org/doi/10.1037/a0015798. 19968428 10.1037/a0015798 PMC3277857

[bib15] Otto, T. U., Öğmen, H., & Herzog, M. H. (2010). Attention and non-retinotopic feature integration. *Journal of Vision,* 10(12), 8, 10.1167/10.12.8. PMC3248829 21047740

[bib16] Plomp, G., Mercier, M. R., Otto, T. U., Blanke, O., & Herzog, M. H. (2009). Non-retinotopic feature integration decreases response-locked brain activity as revealed by electrical neuroimaging. *Neuroimage,* 48(2), 405–414, 10.1016/j.neuroimage.2009.06.031. 19540924

[bib17] Scharnowski, F., Rüter, J., Jolij, J., Hermens, F., Kammer, T., & Herzog, M. H. (2009). Long-lasting modulation of feature integration by transcranial magnetic stimulation. *Journal of Vision,* 9(6), 1, https://psycnet.apa.org/doi/10.1167/9.6.1. 19761292 10.1167/9.6.1

[bib18] Sergent, C., Wyart, V., Babo-Rebelo, M., Cohen, L., Naccache, L., & Tallon-Baudry, C. (2013). Cueing attention after the stimulus is gone can retrospectively trigger conscious perception. *Current Biology,* 23(2), 150–155, 10.1016/j.cub.2012.11.047. 23246406

[bib19] Sergent, C. (2018). The offline stream of conscious representations. *Philosophical Transactions of the Royal Society B: Biological Sciences,* 373(1755), 20170349, 10.1098/rstb.2017.0349. PMC6074088 30061463

[bib20] Taylor, M., & Creelman, C. D. (1967). PEST: Efficient estimates on probability functions. *The Journal of the Acoustical Society of America,* 41(4A), 782–787, 10.1121/1.1910407.

[bib21] Thibault, L., Van den Berg, R., Cavanagh, P., & Sergent, C. (2016). Retrospective attention gates discrete conscious access to past sensory stimuli. *PLoS One,* 11(2), e0148504, 10.1371/journal.pone.0148504. 26863625 PMC4749386

[bib22] Vogelsang, L., Drissi-Daoudi, L., & Herzog, M. H. (2023). Processing load, and not stimulus evidence, determines the duration of unconscious visual feature integration. *Communications Psychology,* 1(1), 8, 10.1038/s44271-023-00011-2. PMC11041769 38665247

